# Exploring the multifaceted roles of GLP-1 receptor agonists; a comprehensive review

**DOI:** 10.3389/fcdhc.2025.1590530

**Published:** 2025-07-10

**Authors:** Bisma Fatima Hammad, Nimrah Zafar, Muneeb Ullah, Syeda Jazilah Faisal, Fizzah Iftikhar, Haadia Waheed, Muhammad Waleed Muzaffar, Khadija Ahmed, Faz Ashraf, Komal Zahid, Maimoona Akhtar, Mohammed Mahmmoud Fadelallah Eljack

**Affiliations:** ^1^ Hammad Department of Medicine, Liaquat National Hospital and Medical College, Karachi, Pakistan; ^2^ Department of Medicine, Dow University of Health Sciences, Karachi, Pakistan; ^3^ Department of Medicine, Saidu Medical College, Swat, Pakistan; ^4^ Department of Medicine, Dow Medical College, Karachi, Pakistan; ^5^ Department of Medicine, Karachi Medical and Dental College, Karachi, Pakistan; ^6^ Department of Medicine, Dow International Medical College, Karachi, Pakistan; ^7^ Department of Medicine, Niazi Medical and Dental College, Sargodha, Pakistan; ^8^ Department of Medicine, Rashid Latif Medical College, Lahore, Pakistan; ^9^ Department of Medicine, Women Medical and Dental College, Abbottabad, Pakistan; ^10^ Community Department, University of Bakht Alruda, Ad Duwaym, Sudan

**Keywords:** glucagon-like peptide-1, type 2 diabetes, endothelial function, obesity, metabolic syndrome

## Abstract

Traditionally, Glucagon-like peptide-1 (GLP-1) receptor agonists (GLP-1RAs), a pivotal class of drug, mimics the actions of endogenous Glucagon-like peptide-1, which have been found to be remarkable in the treatment of type 2 diabetes alongside other comorbidities. GLP-1 receptors being widely available in the different organs and tissues such as the brain, lung, pancreas, stomach, heart, and endometrium has explained the broader therapeutic application of GLP-1RA. The recent studies have explored the physiological effects of GLP-1RA on body organs, establishing them as a potential therapeutic option for a wide range of diseases. Activation of GLP-1 receptors contribute to regulation of blood glucose levels, weight management, cardiovascular health, and potential neuroprotection, while also having a positive influence on musculoskeletal health. This review has emphasized the expanded role of GLP-1RA by highlighting the most significant and notable studies. While GLP-1RA has proven clinical efficacy, the need for more comprehensive studies, to ensure their long-term safety, is essential to optimize their therapeutic role and improve patient outcomes on a global scale. Addressing the significant gap for research on cost effectiveness of these drugs is also crucial for their accessibility in comparison to other drugs. Nevertheless, the limited data available calls for a platform for future research to carry out the expanded therapeutic effects of GLP-1RA.

## Graphical Abstract

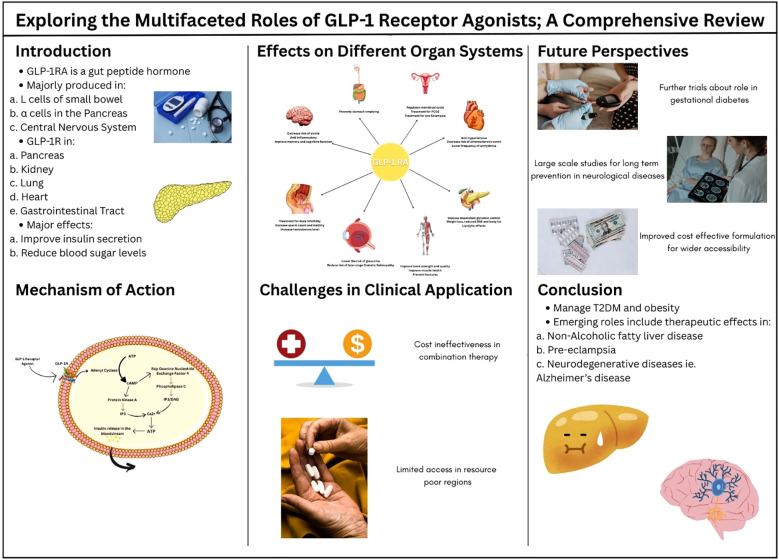

## 1 Introduction

Glucagon-like peptide-1 (GLP-1) is a gut peptide hormone that functions as an incretin that plays a key role in controlling satiety and various other physiological functions. It is majorly found circulating in the blood as GLP-1(7-36) amide that is produced primarily from three tissues of the human body: enteroendocrine L cells in the distal small bowel, α cells in the pancreas, and the central nervous system (1, 2). GLP-1 acts by coupling with the GLP-1 receptor (GLP-1R), which is a 463 amino acid hepta helical G- protein coupled receptor. This receptor is found on pancreatic α and β cells has many effects, a few detailing, regulating glucose levels in the body, improving myocardial function and cardiac output, and delaying gastric emptying.GLP-1 interacts with GLP-1R resulting in the glucose-dependent release of insulin, lowering the plasma glucose levels in the body. In addition, GLP-1 indirectly inhibits glucagon secretion by stimulating release of insulin and somatostatin or by directly engaging with GLP-1R found on α cells of the pancreas (2). GLP-1R are also found in smooth muscle cells in the walls of arteries in kidneys and lungs, myocytes of the sinoatrial node of the heart, and Brunner’s glands of gastrointestinal tract (3). The wide distribution of GLP-1R underscores the variety and significance of its biological functions. Moreover GLP-1R agonists (GLP-1RAs) interact with GLP-1R inducing similar effects as that of GLP-1.

Surprisingly, it took approximately 20 years from the discovery of GLP-1 to the authorized approval of worldwide utilized GLP-1 RAs. Due to the rapid renal clearance of small peptides like GLP-1, along with its deactivation by dipeptidyl peptidase 4 (DPP4), development of stable peptides, with an increased resistance to DPP4 was required (4). In addition, the side effects recorded in clinical trials of GLP-1RAs development programs, nausea and vomiting, prolonged the launch of the drugs (3, 4) Liraglutide and semaglutide are two of the most potent GLP-RAs. Liraglutide was selected as the foremost GLP-1 RA drug to be used for once daily dosing due to its high receptor potency and pharmacokinetics (5). GLP-1 and GLP-1RAs can induce many other effects according to the regions where GLP-1 receptors are found. GLP-1RAs are currently used in treatment of cardiovascular diseases, neurological diseases, metabolic dysfunctions, obesity, peri operative procedures, infertility and many more (6).

The origin of GLP-1 and its physiological pathway was discovered in the 1980s by the decoding of nucleotide sequences of mammalian preproglucagons, the precursors of proglucagon (4). The post translational processing of proglucagon resulted in the formation of glucagon and Glicentin-related pancreatic polypeptide (GRPP) (7). Furthermore, when processed in the intestine, proglucagon yields glicentin, which may be catalyzed further to oxyntomodulin (7). Within minutes of food ingestion, more prominently in carbohydrates and fats, L cells found in the small bowel and colon release GLP-1 into the bloodstream. The exact mechanism with which this occurs is unclear, but it is suspected that neural factors play a key role in this mechanism.

When GLP-1 interacts with GLP-1R, this triggers cyclic adenosine monophosphate (cAMP) formation and initiates subsequent pathways (2). A comprehensive understanding of the systemic effects of GLP-1 receptor agonists is crucial because these medications impact multiple organ systems beyond their primary role in glucose regulation. GLP-1 RAs not only improve insulin secretion and reduce blood sugar levels, but they also affect appetite control, weight management, and cardiovascular health. A deeper knowledge of their broad effects can optimize their therapeutic use in treating conditions like type 2 diabetes, obesity, and heart disease, while minimizing side effects and improving patient care. This narrative review aims to explore the effects of GLP-1RAs on the multiple systems of the body.

## 2 Methodology

A structured literature search was conducted using PubMed/MEDLINE and Google Scholar, with the Boolean search string: (“GLP-1 receptor agonist” OR “GLP-1RA”) AND (“multifaceted roles” OR “pleiotropic effects”). The initial search yielded 1,929 records (PubMed: 29; Google Scholar: 1,900). After removing 420 duplicates and excluding 23 articles prior to screening, 1,509 records remained for title and abstract review. Of these, 1,200 studies were excluded due to lack of relevance or unsuitable study design. The remaining 309 articles underwent full-text assessment. An additional 202 articles were excluded for the following reasons: irrelevant study population (n=60), unrelated interventions (n=90), insufficient or incomplete data (n=52), and language limitations (non-English). Ultimately, 107 studies were included in this narrative review.

**Figure 1:**
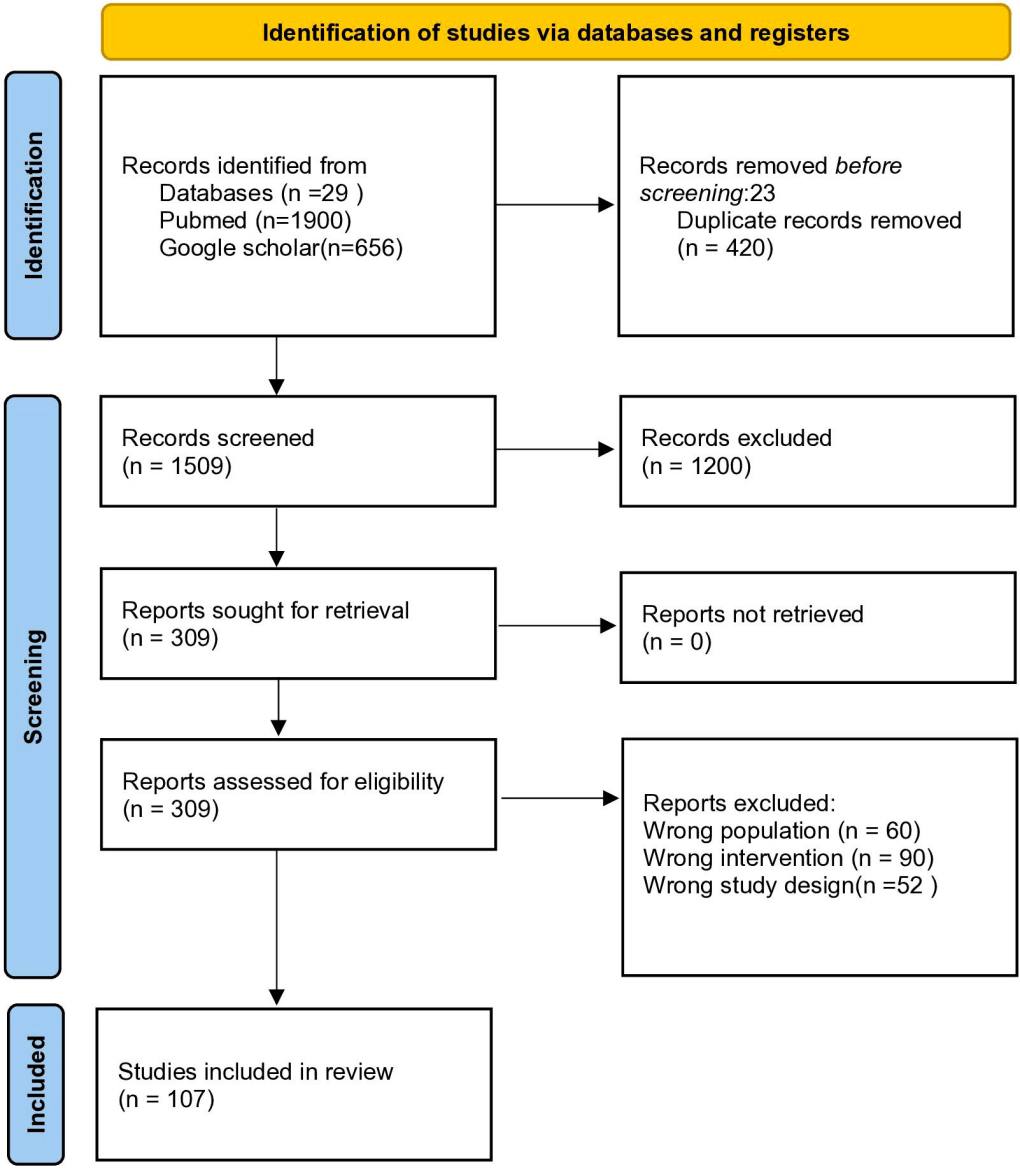
PRISMA-style flow diagram illustrating the literature selection process for this narrative review on the multifaceted roles of GLP-1 receptor agonists.

The selection process is illustrated in the flow diagram (**Figure 1**).

## 3 Mechanism of action

GLP-1RA mimics the role of GLP-1, a hormone released by the intestines after consumption of glucose-rich foods (8). GLP-1RA is administered subcutaneously to bypass the digestive system, except for semaglutide which is given orally (9). It binds to the GLP-1 receptor in various metabolic sites, mainly the pancreas where it stimulates them for the release of insulin by the β cells. As a result, glucose uptake by the intestine and the muscles increases (10). It also plays a role in inhibiting β cell apoptosis, which is common in conditions like Type 2 Diabetes Mellitus (T2DM) and enhancing their growth (11). Moreover, it inhibits glucagon release from the α cells of the pancreas and promotes slow stomach emptying, hence giving the feeling of fullness and reduces body weight (12).

**Figure 2:**
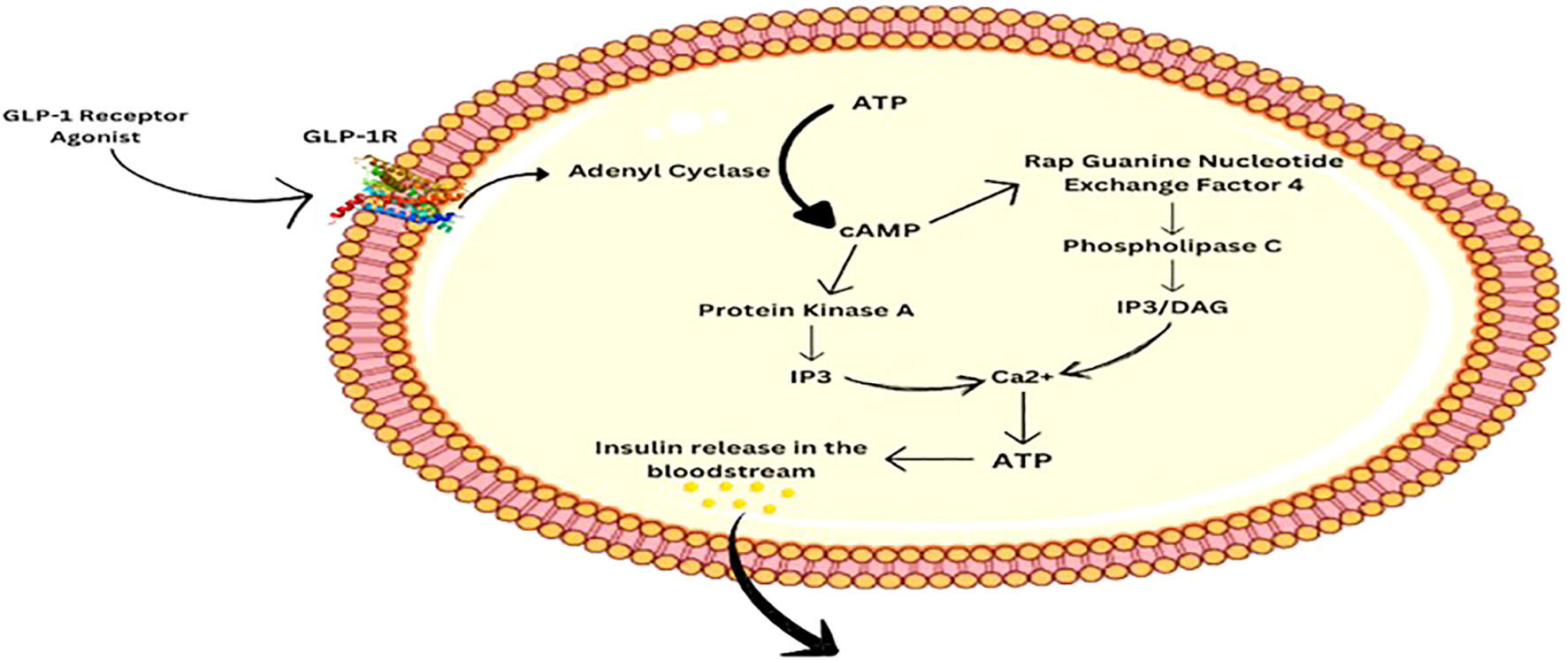
The GLP-1RA pathway involved in insulin release.

GLP-1R is a G protein-coupled receptor. When the hormone or the receptor agonist binds to it, a series of cascade effects occur, eventually leading to the release of Ca2+. The binding of GLP-1RA to GLP-1R activates adenyl cyclase, which facilitates the conversion of ATP to cAMP. cAMP then activates Protein Kinase A (PKA) and Rap Guanine Nucleotide Exchange Factor 4 (RGNEF4), both of which function for rising intercellular Ca2+ levels. PKA causes membrane depolarization and eventually Ca2+ release via the Inositol Triphosphate pathway (IP3). RGNEF4 aids in Ca2+ release via IP3 as well as the Diacylglycerol (DAG) pathway by activating Phospholipase C. Elevated levels of calcium in the intracellular space promote ATP production, which then facilitates the release of Insulin into the bloodstream (6, 10) as shown in **Figure 2**. Eventually, it leads to uptake of glucose by the body cells and decreasing the level in the blood.

## 4 Efficacy of GLP1 RA

**Figure 3:**
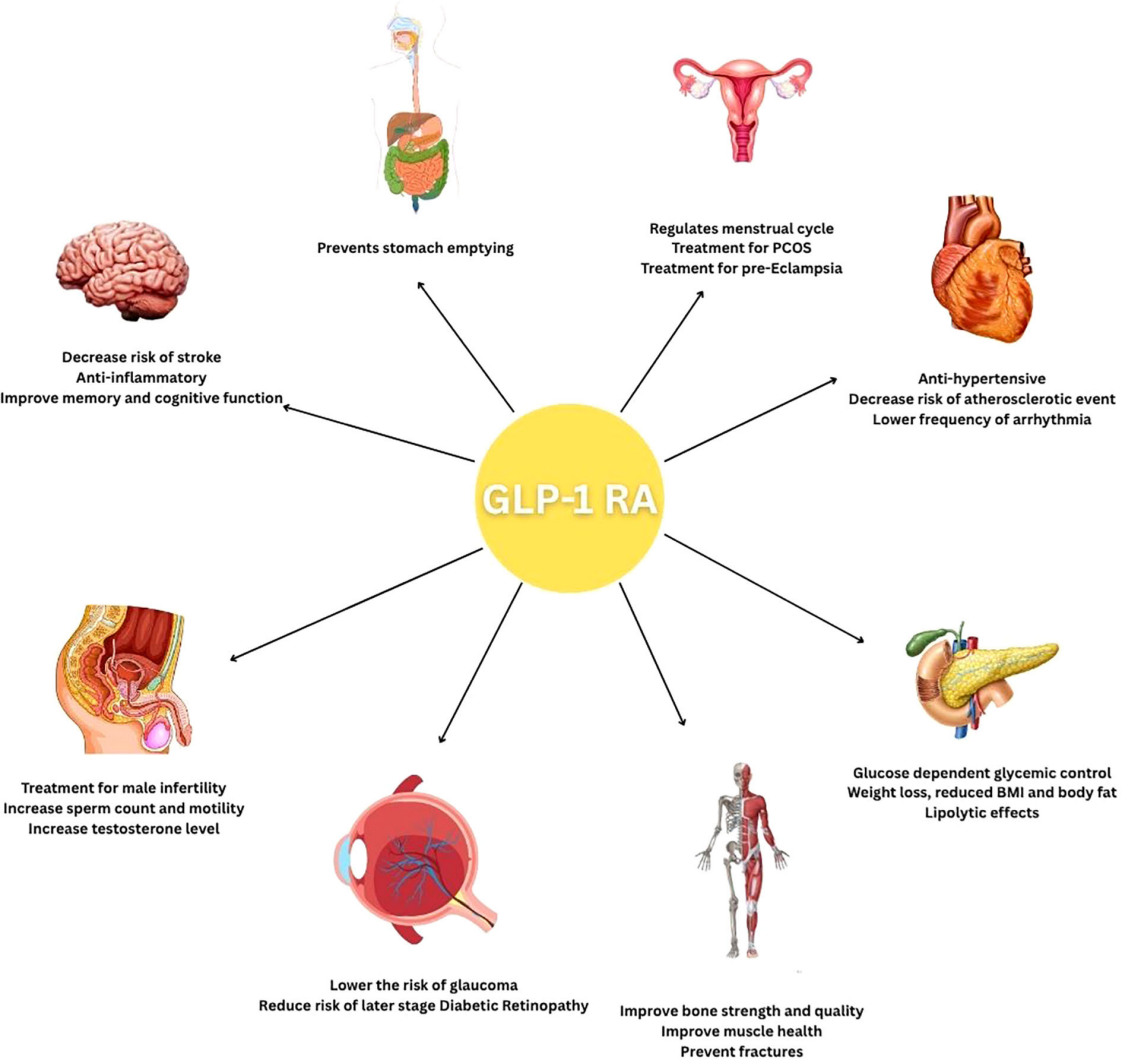
A cartoon summarizing the disease groups and organs in which GLP-1 receptor agonists are expected to be effective.

GLP-1 receptor agonists (GLP-1RAs) have demonstrated significant clinical benefits that extend well beyond glycemic control in type 2 diabetes. Emerging evidence from key clinical trials highlights their multifaceted roles across various physiological systems, including cardiovascular, renal, hepatic, and neurological domains, as shown in **Figure 3**. These agents exhibit anti-inflammatory, cardioprotective, and neuroprotective effects, suggesting a broader therapeutic potential.

**Table 1** summarizes major clinical trials evaluating the efficacy of GLP-1RAs across different organ systems, illustrating the expanding scope of their clinical utility.

**Table 1:** Comparative summary of recent key clinical trials evaluating GLP-1 RA efficacy.

Study	Year	GLP-1 RA	Population	Comparator	Clinical endpoints	Key findings
LEADER (13)	2016	Liraglutide	Patients with type 2 diabetes and high cardiovascular risk (n=9,340)	Placebo plus standard care	Primary: Major adverse cardiovascular events (MACE) including cardiovascular death, nonfatal myocardial infarction, and nonfatal stroke. Secondary: All-cause mortality, renal outcomes, and microvascular complications.	Liraglutide significantly reduced the risk of MACE by 13% compared to placebo. The risk of cardiovascular death was reduced by 22%, and all-cause mortality decreased by 15%. Renal outcomes also improved, with a 22% reduction in nephropathy events. There was no statistically significant difference in diabetic retinopathy complications between the liraglutide and placebo groups.
SUSTAIN- 6 (14)	2016	Semaglutide	Patients with type 2 diabetes at high cardiovascular risk (n=3,297)	Placebo	Primary: Major adverse cardiovascular events (MACE) comprising cardiovascular death, nonfatal myocardial infarction, and nonfatal stroke. Secondary: Individual components of MACE, all-cause mortality, nephropathy, and retinopathy complications.	Semaglutide significantly reduced the risk of MACE by 26% compared to placebo (hazard ratio [HR], 0.74; 95% confidence interval [CI], 0.58 to 0.95; P<0.001 for noninferiority). The incidence of nonfatal stroke was notably lower in the semaglutide group (HR, 0.61; 95% CI, 0.38 to 0.99). Semaglutide also reduced the risk of new or worsening nephropathy by 36% (HR, 0.64; 95% CI, 0.46 to 0.88). However, there was an observed increase in diabetic retinopathy complications in the semaglutide group (HR, 1.76; 95% CI, 1.11 to 2.78), primarily among patients with pre-existing retinopathy and those experiencing rapid improvements in glycemic control.
EXSCEL (15)	2017	Exenatide (once-weekly)	patients with type 2 diabetes, with or without previous cardiovascular disease (n = 14,752)	Placebo plus usual care	Primary: Major adverse cardiovascular events (MACE) comprising cardiovascular death, nonfatal myocardial infarction, and nonfatal stroke. Secondary: All-cause mortality, hospitalization for heart failure, and other cardiovascular outcomes.	Exenatide did not significantly reduce the incidence of MACE compared to placebo (HR 0.91; 95% CI, 0.83–1.00; P=0.06). All-cause mortality was similar between the exenatide and placebo groups. The safety profile of exenatide was consistent with previous studies, and no new safety concerns were identified.
HARMONY Outcomes (16)	2018	Albiglutide	Patients with type 2 diabetes and established cardiovascular disease (n = 9,463)	Placebo	Primary:: Major adverse cardiovascular events (MACE: cardiovascular death, nonfatal myocardial infarction, nonfatal stroke) Secondary: Individual MACE components, all-cause mortality, heart failure, and safety	Albiglutide significantly reduced the incidence of MACE by 22% compared to placebo (HR 0.78; 95% CI, 0.68–0.90; P<0.0001). The reduction was largely driven by a significant decrease in nonfatal myocardial infarction. Cardiovascular and all-cause mortality were not significantly different between groups. Albiglutide was well tolerated with a safety profile consistent with previous GLP-1 RAs.
REWIND (17)	2019	Dulaglutide (1.5 mg weekly)	Patients with type 2 diabetes, including those with and without established cardiovascular disease (n = 9,901)	Placebo plus standard care	Primary: Major adverse cardiovascular events (MACE) comprising cardiovascular death, nonfatal myocardial infarction, and nonfatal stroke. Secondary: Individual components of MACE, all-cause mortality and safety assessments.	Dulaglutide significantly reduced the incidence of MACE by 12% compared to placebo (hazard ratio [HR] 0.88; 95% confidence interval [CI], 0.79–0.99; P=0.026). The reduction in MACE was consistent across subgroups, including those without established cardiovascular disease. All-cause mortality was numerically lower but did not reach statistical significance (10.8% vs 12.0%; HR 0.90; 95% CI, 0.80–1.01; P=0.067). Gastrointestinal adverse events were more frequent in the dulaglutide group (47.4%) compared to placebo (34.1%; P<0.0001). Dulaglutide was otherwise well tolerated with no new safety concerns.
AMPLITUDE-O (18)	2021	Efpeglenatide	patients with type 2 diabetes and either a history of cardiovascular disease or current kidney disease plus at least one other cardiovascular risk factor (n = 4,076)	Placebo plus standard care	Primary: Major adverse cardiovascular events (MACE: cardiovascular death, nonfatal myocardial infarction, nonfatal stroke) Secondary: Composite renal outcomes (e.g., sustained decrease in estimated glomerular filtration rate, progression to end-stage kidney disease, or death from renal causes), all-cause mortality, safety assessments	Efpeglenatide significantly reduced the risk of MACE by 27% compared to placebo (hazard ratio [HR], 0.73; 95% confidence interval [CI], 0.58–0.92; P<0.001 for noninferiority; P=0.007 for superiority). The composite renal outcome was also significantly reduced (HR, 0.68; 95% CI, 0.57–0.79; P<0.001). Gastrointestinal adverse events were more frequent in the efpeglenatide group, but overall safety was consistent with the GLP-1 receptor agonist class.
STEP 1 (19)	2021	Semaglutide 2.4 mg weekly	adults with overweight or obesity (BMI ≥30 or ≥27 with ≥1 weight-related comorbidity), without diabetes (n = 1,961)	Placebo + lifestyle intervention	Primary: Percent change in body weight at 68 weeks	Semaglutide led to a 14.9% mean weight loss vs 2.4% with placebo (mean difference: −12.4 percentage points; P<0.001).
Liraglutide Pediatric Obesity Trial (20)	2024	Liraglutide 3.0 mg daily	Children aged 6 to <12 years with obesity	Placebo + lifestyle intervention	Primary: Change in BMI standard-deviation score (SDS) at 56 weeks Secondary: Proportion achieving ≥5% BMI reduction; changes in body weight, waist circumference, cardiometabolic risk factors; safety	Liraglutide led to a greater reduction in BMI SDS compared to placebo at 56 weeks. A higher proportion of participants in the liraglutide group achieved a ≥5% reduction in BMI. Improvements were also observed in body weight and waist circumference. Gastrointestinal adverse events were more common in the liraglutide group; however, the overall safety profile was consistent with previous findings in adolescents.
SURMOUNT-5 (21)	2024	Tirzepatide (15 mg weekly)	Adults with obesity (BMI ≥30) or overweight (BMI ≥27) with at least one weight-related comorbidity, without diabetes (n = 751)	Semaglutide (2.4 mg weekly)	Primary: Percent change in body weight at 72 weeks Secondary: Proportion achieving ≥5%, ≥10%, ≥15%, and ≥20% weight loss; changes in waist circumference, cardiometabolic risk factors; safety	Tirzepatide resulted in greater weight loss compared to semaglutide at 72 weeks. A higher proportion of participants in the tirzepatide group achieved ≥5%, ≥10%, ≥15%, and ≥20% weight loss. Improvements were also observed in waist circumference and cardiometabolic risk factors. Gastrointestinal adverse events were more common in both groups, with a higher incidence in the tirzepatide group.

### 4.1 Central nervous system effects

GLP-1R is distributed throughout various regions in the brain, with the highest concentrations being found in the hippocampus and cerebral cortex, areas mainly responsible for cognitive function, memory improvement, and learning (10). The receptors are also predominantly found in the brainstem, hypothalamus, cerebellum, and limbic system (22), as well as in microglia and astrocytes (23). The GLP-1RA needs to cross the Blood-Brain Barrier (BBB) to act on the brain and exert neuroprotective effects (12, 22). Drugs like exenatide and lixisenatide have a greater penetration ability as compared to liraglutide (10).

GLP-1RA plays a key role in promoting CNS health in conditions like T2DM and lowering the risk of stroke (23). After crossing the BBB, the GLP-1RA binds to the GLP-1R and mimics the action of the GLP-1 hormone, stimulating the intracellular accumulation of cAMP, which via the action of PKA/RGNEF4 results in neuroprotective effects (10). However, the exact pathway is still unknown.

In the brain, GLP-1RAs perform anti-inflammatory, antioxidant, and anti-apoptotic functions (12). By reducing inflammation, Liraglutide protects the neuron from inflammatory damage and stimulates neural cell and stem cell proliferation (22), as well as neurite outgrowth, which is important for synaptic functions that play a key role in improving memory in neurodegenerative diseases like Alzheimer’s Disease (AD) and Parkinson’s Disease (PD). GLP-1RA also minimizes oxidative stress, which is a significant factor in the progression of neurodegenerative diseases (24). They attain this by inhibiting the intracellular accumulation of Reactive Oxygen Species (ROS) and increasing the expression of glutathione peroxidase and superoxide dismutase, thus improving the adverse cellular changes induced by ROS. Furthermore, the expression of Brain-Derived Neurotrophic factor (BDNF) is enhanced, which is essential for the proper functioning of brain neurons (22). It inhibits neural apoptosis and increases survival and regeneration, thus forming new connections, ultimately contributing to improved cognitive function (12, 25)

Appetite regulation is a common function of many GLP-1RAs, which act on the hypothalamus- the center of hunger and energy balance- and promote feelings of fullness, eventually leading to weight loss (26). Toxic accumulation of Amyloid β protein, commonly associated with Parkinson’s Disease (27), disrupts Long Term Potentiation (LTP) (24); however, it can be reversed by GLP-1RA like Exendin-4 (Ex-4), leading to improvement in cognitive functions and memory (22). Additionally, Ex-4 also inhibits MPTP (1-methyl-4-phenyl-1,2,3,6-tetrahydropyridine) toxin, mainly responsible for the destruction of dopaminergic neurons in Substantia Nigra in the brain, which is the main cause of Parkinson’s Disease (24). By improving insulin signaling, GLP-1RAs reduce insulin resistance, leading to improvements in the pathophysiology of Alzheimer’s Disease and Parkinson’s Disease (6). They are not only protective in the early stages of Alzheimer’s Disease but can also reverse key pathophysiological features like amyloid β protein and tau hyperphosphorylation, thereby protecting the neurons from further damage (10). Liraglutide also has antidepressant effects, as seen in many patients with neurodegenerative diseases, by preventing the loss of insulin receptors (28). Exenatide has shown effectiveness in reducing motor defects in Parkinson’s Disease by preserving dopaminergic neurons and enhancing neurotransmitter function, thus improving the overall outcome (29).

### 4.2 Cardiovascular system effects

GLP-1 receptor agonists have a very close connection with cardiovascular outcomes and prevention of many diseases. It has been proven that glucagon-like peptide-1 receptor (GLP-1R) agonists control blood glucose concentrations through a multitude of mechanisms, such as increased satiety, slow stomach emptying, decreased glucagon secretion, and increased insulin synthesis/secretion. Patients with T2DM and a high cardiovascular risk might reduce their risk of atherosclerotic events via the use of GLP-1RA (30). GLP-1RAs lower plasma lipid levels and lower blood pressure, both of which contribute to a reduction of atherosclerosis and cardiovascular disease (31). The main causes of coronary heart disease, end-stage renal failure, acquired blindness from central retinal artery occlusion (32). and various neuropathies are accelerated atherosclerosis and microvascular problems. These elements could be contributing to the high mortality rates and disabilities seen in those with diabetes (33).

Many physicians and researchers have become interested in the potential cardiovascular advantages of GLP-1 and GLP-1-based medication since the discovery of GLP-1Rs on cardiomyocytes. Small-scale clinical trials revealed that GLP-1 and GLP-1-based medicines may have positive effects on low-grade inflammation, blood pressure, cholesterol, and microcirculation (34). More significantly, GLP-1RAs have been shown in large, randomized CV outcome trials (CVOTs) to lower CVD in patients with type 2 diabetes (34). Liraglutide and Semaglutide most effectively lower Hb1Ac and regulate glucose thereby reducing the risk of type 2 diabetes which is a main cause of cardiovascular problems. Liraglutide has a half-life of 13 hours in humans and semaglutide has a half-life of 160 hours (35). Recent cardiovascular outcome trials involving GLP-1 receptor agonists, especially liraglutide and semaglutide, have shown a significant reduction in major adverse cardiovascular events (MACE) among patients with type 2 diabetes at high risk for cardiovascular disease (14, 36). These effects are thought to result from anti-atherosclerotic actions, possibly mediated by anti-inflammatory mechanisms. Humans treated with liraglutide have been shown to have reduced inflammation, which is linked to decreased levels of TNF-α, IL-1β, IL-6, and CD163, a cluster of differentiation (37). Atherosclerosis is a consequence that diabetic people may experience as a result of injury to their vascular smooth muscle cells (VSMCs) (38). GLP-1RAs help improve endothelial damage and slow the advancement of cardiovascular disorders. They exert direct positive impacts on the coronary vascular endothelium, such as oxidative stress reduction and increase in nitric oxide, which at least partially account for their antihypertensive and antiatherosclerotic properties (39). LDL and C-reactive protein (CRP) lipid distribution in atherosclerosis were improved by liraglutide with metformin in newly diagnosed diabetic patients following standard statin therapy (40).

Due to their various modes of action, which include vasodilation, natriuresis, glucose and weight control, and direct cardioprotective effects, GLP-1 receptor agonists have therapeutic potential for treatment of heart failure (41). Ischemic heart disease, dilated cardiomyopathy, valvular heart disease, and hypertensive heart disease are among the main causes of heart failure (42).

Obesity is a major cause of cardiovascular diseases. It is linked to elevated levels of fibrinogen and C-reactive protein, diabetes, insulin resistance, dyslipidemia, and hypertension, all of which raise the risk of CVD events (43). Obesity has been demonstrated to raise the risk of high blood pressure in addition to CVD (43). Treating obesity can be greatly beneficial for the prevention of CVD. Agents that have been shown to be remarkably effective in promoting weight loss and improving metabolic outcomes include liraglutide, semaglutide, and tirzepatide. These agents have been supported by important clinical trials, including Satiety and Clinical Adipose Liraglutide Evidence (SCALE), Semaglutide Treatment Effect in People with Obesity (STEP) program trials, and Tirzepatide Once Weekly for the Treatment of Obesity (SURMOUNT-1) (44). Hence, these agents can prevent obesity, thereby reduce CVD.

It is less evident how GLP-1 agonists directly affect arrhythmias, some studies suggest they may reduce frequency of arrythmias because their favorable effects on metabolism and over all cardiovascular health. Arrhythmia risk may be lowered, by better glycemic control and a decrease in obesity-related cardiovascular risk factors. It is less clear how GLP-1 receptor agonists (GLP-1 RAs) directly influence arrhythmia risk; however, emerging evidence suggests a potential protective effect. Some studies indicate that GLP-1 RAs may help reduce the frequency of arrhythmias due to their beneficial impact on metabolic parameters and overall cardiovascular health (45) A recent systematic review and meta-regression analysis found that semaglutide significantly reduced the incidence of new-onset atrial fibrillation in patients with type 2 diabetes (46). These findings highlight the need for further investigation into the antiarrhythmic potential of GLP-1 RAs.

One of the main causes of the excess cardiovascular risk in diabetics is the coexistence of hypertension. Through a diuretic mechanism and other effects on the kidneys, GLP-1 can lower blood pressure. On a different level, GLP-1RAs reduce the concentration of angiotensin II (Ang II), which lowers arterial hypertension in T2DM and improves systemic insulin sensitivity (31). Regarding the enhancement of hard cardiovascular outcomes while preserving cardiovascular safety, GLP-1RAs have proved encouraging efficacy.

### 4.3 Gastrointestinal effects

The gastrointestinal (GI) tract is greatly impacted by GLP-1RAs. They prevent the stomach from emptying, and this helps in regulating postprandial glucose levels. Higher doses cause more inhibition in this dose-dependent effect. Tachyphylaxis, which reduces gastrointestinal side symptoms such nausea and vomiting, might result with prolonged use. GLP-1RAs work by way of neural pathways, specifically the vagus nerve. On the other hand, gastric emptying is not considerably impacted by glucose-dependent insulinotropic polypeptide (GIP), emphasizing the distinct functions of these incretins in GI function (47).

A study examined the potential advantages and disadvantages of using GLP-1RAs to help obese but non-diabetic patients lose weight. With 8,847 participants over eight randomized controlled trials (RCTs), it was shown that 375 out of 1,000 people lost 10% of their body weight over the course of two years, with a net benefit probability of 0.91 in the second year and 0.97 in the first. The study did, however, also highlighted several negative side effects, including constipation (118 occurrences), diarrhea (100 events), and abdominal pain (41 events per 1,000) (48).

Abdominal pain, nausea, vomiting, and diarrhea are among the gastrointestinal adverse effects linked to GLP-1 RAs. These effects are influenced by factors such as dosage and background medications. A systematic analysis reported that using metformin alongside GLP-1RAs in type 2 diabetes management increases the risk of nausea and vomiting. Additionally, lixisenatide was associated with fewer gastrointestinal symptoms compared to exenatide, while long-acting GLP-1RAs generally caused less nausea and vomiting but more diarrhea than short-acting agents (49). Among individual drugs, liraglutide has been noted to carry a relatively higher risk of nausea and vomiting. Nevertheless, compared to placebo, none of the GLP-1RAs significantly increased the risk of serious gastrointestinal complications (50).

### 4.4 Metabolic effects

GLP-1RA are emerging as highly versatile drugs, as their unraveling features now adds to a broader potential in future. GLP-1RA has shown explicit effects in controlling blood glucose levels. Unlike sulfonylureas, which stimulate insulin secretion independently of blood glucose and can lead to hypoglycemia, GLP-1RAs improve glycemic control in a glucose-dependent manner, thereby minimizing the risk of hypoglycemia. They achieve this by signaling a cascade consisting of cAMP and protein kinase A and the closure of K ATP gates, induced by PKA cycle, marks the initiation of glucose dependent insulin secretion into the bloodstream (51).

Also, GLP-1 RA seems to regulate glucose homeostasis by exhibiting glucagonostatic effects meaning suppression of glucagon by acting on pancreatic α cells, which in turn reduce hepatic production of glucose (52). Interestingly, GLP-1 receptors are also found on pancreatic delta cells, producing somatostatin, an inhibitory hormone, further dampening glucagon secretion (53). A study conducted on rodents has also revealed that GLP-1RAs can exert glucagonotropic effects depending on glucose levels, adding complexity to their role in glucagon regulation (52). Moreover, these drugs promote the neogenesis of pancreatic β cells by enhancing B cell proliferation and reducing apoptosis, contributing to the preservation and expansion of insulin-secreting cells (54).

Liraglutide has been reported to lower the BMI by approximately 5% (55) and semaglutide with an average of 15% compared to a placebo (56). A recent comparison of GLP-1RAs versus placebo in both pediatric and adult populations demonstrated the significant efficacy of GLP-1RAs in promoting weight loss, highlighting their potential across diverse age groups (57).

Furthermore, a statistical analysis was executed to unearth the potential of lixisenatide, both alone and in combination with insulin, on gastric emptying. The study confirmed that lixisenatide exerts a more pronounced effect on postprandial glycemic variations with a modest effect on fasting glucose levels. This is largely attributed to glucagon suppression and slow gastric emptying. In contrast, insulin primarily affects fasting glucose levels with minimal influence on post meal glucose levels and no relation to gastric emptying (58).

The improved regulation of glycemia by GLP-1RA, along with their ability to reduce lipid synthesis, enhanced beta oxidation of free fatty acids and autophagy of fat cells contributes to their lipid modulation properties (59). It has been proposed that GLP 1 exerts a lipolytic effect by breaking down triglycerides into free fatty acids in pancreatic β cells. This process aids in ATP production, which subsequently stimulates insulin secretion (60).

Oral semaglutide has been found to improve fasting blood glucose levels in a trial conducted over a 12 week period with improved levels of triglycerides, very low-density lipoprotein (VLDL), low- density lipoprotein (LDL) and apolipoprotein B48 (ApoB48) and no change in high density lipoprotein (HDL) (61). Furthermore, type 2 diabetes has been linked to an increased risk of visceral fat, including epicardial adipose tissue (EAT), which expresses GLP-1 and GLP-2 receptors. In a study of 80 obese people with type 2 diabetes receiving semaglutide and dulaglutide, a 20% reduction in the thickness of EAT was observed (62) One meta-analysis demonstrated the cardioprotective effects of GLP-1 RAs, showing reductions in blood pressure and lipid profile, with a significant increase in HDL levels (63).

### 4.5 Musculoskeletal effects

T2DM raises the risk of fractures, poor bone repair, and other factors that lead to brittle bones (64). GLP-1RAs have demonstrated encouraging positive benefits on bone strength and quality. The blood flow to bones is vital for skeletal health, and GLP-1RAs may improve it, while the precise processes are not entirely known. This is especially important for older people with type 2 diabetes, as they have an increased risk of osteoporosis and fractures (65). GLP-1 receptor analogs, such as exenatide, have a beneficial effect on bone health in women with type 2 diabetes who have gone through menopause (66). The anti-resorptive effects of the therapy were demonstrated by lower RANK and RANKL levels and increased osteoprotegerin (OPG) levels. Significant weight reduction was seen, but bone mineral density (BMD) did not change. This implies that GLP-1RAs may support BMD maintenance during weight loss, providing a dual advantage in the management of diabetes (67). GLP-1R agonists have shown potential benefits in muscle health by decreasing muscle atrophy and inflammation, as well as improving muscle microvasculature and endurance (68). They suppress muscle atrophic factors like myostatin and promote myogenic factors, which can enhance muscle protein synthesis and mitochondrial function. These effects suggest that GLP-1R agonists may mitigate the muscular deleterious effects associated with conditions like idiopathic inflammatory myopathy (68). GLP-1RAs are essential for preventing fractures in people with T2DM because they greatly increase bone mass, trabecular and cortical architecture, and total bone strength. They counteract the imbalance between bone production and resorption that is typical in type 2 diabetes by promoting bone formation and enhancing blood supply to bones. Although skeletal effects are beneficial in preclinical investigations, clinical evidence on fracture risk is still uncertain. To better understand these connections and evaluate GLP-1RAs’ potential as a therapeutic approach for treating skeletal frailty in diabetes patients, especially the elderly more extensive long-term research is required (65). GLP-1RAs stimulate the production of new bone while blocking its resorption, increasing BMD and improving bone quality. Nutrient absorption may be hampered by gastrointestinal side effects, which would exacerbate their impact on musculoskeletal health. To fully understand GLP-1RAs’ effects on bone metabolism in a range of patient groups, particularly regarding osteoporosis and fracture risk management, more randomized controlled studies are necessary (69).

A study that investigated the effects of insulin and GLP-1RAs (exenatide and dulaglutide) on BMD over a 52-week duration gathered 70 individuals with type 2 diabetes. When compared to a placebo, exenatide dramatically raised BMD at the femoral neck and whole hip. Femoral neck BMD decreased after using dulaglutide, although not as much as when using a placebo. Moreover, insulin glargine increased BMD. The study emphasizes the necessity of more investigation to comprehend the processes behind these impacts on fracture risk and bone metabolism (70).

It has been demonstrated that GLP-1 agonists significantly increase the mass of the femur and vertebrae (71). Most of the clinical evidence points to GLP-1RAs as having no discernible effect on BMD or fracture rates, indicating that they have a neutral effect on bone health. For people with diabetes who are more likely to have a bone fracture, this neutrality is essential. Sclerostin (SOST) mRNA levels have been discovered to be lowered by GLP-1, which is known to inhibit the formation of new bone. This decrease could encourage osteoblast development and differentiation, which would benefit the skeleton (69).

In individuals with T2DM, the risk of fractures was assessed using a network meta-analysis and systematic review to determine the relationship between dipeptidyl peptidase-4 inhibitors (DPP-4i), GLP-1 RAs, and sodium-glucose cotransporter-2 inhibitors (SGLT-2i). With a median follow-up duration of 26 weeks, a total of 177 randomized controlled trials involving 165,081 participants were included. Comparison with other antidiabetic medicines like insulin, metformin, sulfonylureas, thiazolidinediones, α -glucosidase inhibitors placebo showed no statistically significant increase in fracture risk for DPP-4i, GLP-1 RAs, or SGLT-2i. The investigation specifically showed that these drugs did not increase the risk of any fractures, confirming their safety profile in clinical practice (72).

### 4.6 Ophthalmological effects

#### 4.6.1 Glaucoma

GLP-1R agonists exhibit anti-inflammatory and neuroprotective properties, suggesting they could also be beneficial in neurodegenerative diseases (73). Glaucoma is one such disease, characterized by the degeneration of retinal ganglion cells (RGCs) and optic nerve atrophy, leading to progressive and permanent vision loss. It is the leading cause of irreversible blindness globally, with projections indicating it could affect over 100 million individuals by 2040 (74). Regardless of the glaucoma type, all current treatments focus on lowering intraocular pressure (IOP) by either reducing aqueous humor production or enhancing its outflow (75).

A recent registry-based case-control study, summarized in **Table 2**, involving 1,961 patients found that the use of GLP-1R agonists is linked to a lower risk of glaucoma (Sterling et al., 2021 (76). This finding strongly encourages further investigation into the potential of agents that enhance GLP-1R signaling as treatments for glaucoma (79).

**Table 2:** Clinical studies elucidating the use of antidiabetics against glaucoma.

Compound	Study type	Study design	Study outcome	References
GLP-1R agonists	Observational	1,961 patients with no baseline glaucoma, glaucoma suspect nor ocular hypertension who newly initiated GLP-1RA treatment, e.g., *semaglutide*, were compared to an unexposed control group.	Reduced the hazard for both a new diagnosis of glaucoma and glaucoma suspect (i.e., angle closure, no damage).	(76)
GLP-1R agonists	Observational, retrospective cohort study	This observational, retrospective cohort study included two cohorts: Cohort A comprised 8,876 patients with newly diagnosed T2DM treated with six different classes of antidiabetic drugs, while Cohort B consisted of 4,161 patients with T2DM who initiated treatment with GLP-1RAs.	The complications of diabetes were reduced with reductions in BMI and HbA1c.	(77)
GLP-1R agonists	Observational cohort study	The study utilized propensity score matching to create comparable cohorts of individuals with obesity and without T2DM, including those new to GLP-1RA treatment and those not receiving glucose-lowering medications. The analysis was conducted using data from the TriNetX Global Network for individuals with an index event between January 1, 2016, and March 31, 2024.	The study evaluated primary outcomes such as adverse events, cardiovascular risks, acute kidney injury, and all-cause mortality over five years. It was found that GLP-1RAs significantly protect individuals with obesity and without T2DM. Improved cardiovascular and renal health from GLP-1RAs may also benefit ocular health, potentially reducing the risk of glaucoma related to metabolic disturbances.	(78)

The use of GLP-1 agonists has been associated with a reduced incidence of glaucoma in diabetic patients (80). A study utilizing a national database found that individuals treated with GLP-1 receptor agonists had a significantly lower risk of developing glaucoma compared to those who were not on these medications. This study, which included around 6,400 patients, employed Cox regression analysis and confirmed that diabetic patients using GLP-1R agonists—such as exenatide, liraglutide, dulaglutide, and semaglutide were less likely to receive a new glaucoma diagnosis (76). A study shows that GLP-1 receptor agonists show a notably reduced incidence of primary open-angle glaucoma (POAG), ocular hypertension, and the requirement for first-line glaucoma treatments compared to metformin in individuals with type 2 diabetes (80). A summary of clinical studies elucidating the use of antidiabetics against glaucoma is given in **Table 2**.

#### 4.6.2 Retinal disease

Diabetic retinopathy (DR) is the most prevalent microvascular complication of diabetes and the leading cause of preventable blindness among working-age individuals (81). Currently, nearly 100 million people globally are affected by DR, and as the prevalence of diabetes continues to rise, this number is expected to grow, posing a significant burden on public health (82).

The secretion of VEGF and IL-6 is crucial in diabetic eye diseases (83). In a study, cells were treated with 30 mM glucose with or without liraglutide (50, 100 nM) for 24 hours, after which VEGF-A and IL-6 levels were measured using ELISA. The level of VEGF-A significantly increased from 75.6 to 138.6 pg/mL in high glucose (HG)-stimulated human retinal endothelial cells (HRECs) but decreased to 102.1 pg/mL and 92.5 pg/mL with 50 nM and 100 nM liraglutide, respectively. Similarly, IL-6 levels in the control, HG, 50 nM liraglutide, and 100 nM Liraglutide groups were 135.2, 256.8, 191.6, and 172.9 pg/mL, respectively. These findings indicate that Liraglutide suppresses the release of VEGF-A and IL-6 in HG-stimulated HRECs (84). Liraglutide. Research indicates that VEGF levels in the serum of patients with DR progressively rise as the disease worsens, highlighting its critical role in the development of DR and its correlation with disease severity. Additionally, factors secreted by retinal cells, like IL-6, promote the proliferation of different retinal cell types, contributing to the formation of new blood vessels and the progression of diabetic retinopathy (84).

A meta-analysis was done which indicates that albiglutide is linked to a higher risk of early-stage DR compared to placebo, while showing a reduced risk of late-stage DR compared to insulin (85) Furthermore, the other six FDA-approved GLP-1RAs do not demonstrate a statistically significant association with DR. To fully understand the differences in the mechanisms and long-term effects of GLP-1RAs on the retina, a combination of clinical trials and mechanism studies is necessary (85). The results from the SUSTAIN 6 trial indicated that patients treated with semaglutide experienced a significantly higher incidence of DR compared to those receiving placebo (86).

### 4.7 Pregnancy-related effects

Although type 2 diabetes and obesity have been effectively treated with GLP-1 receptor agonists, their role in pregnancy and breastfeeding has been a controversial debate due to potential risks to the mother’s health and fetal outcomes (87). Decisions about therapy during nursing should be carefully weighed (88) since each medication taken by a woman in this period has potential adverse effects that are obvious to the fetus. Certain detrimental effects on a child’s health are strongly correlated with maternal obesity and gestational diabetes underscoring the need for a safer and more efficient drug (89).

Since none of the GLP-1RA are approved in pregnancy, a cohort study approved the potency of liraglutide, in early pregnancy, despite its adverse outcomes such as preterm birth, preeclampsia and gestational diabetes mellitus (GDM) (90). These adverse effects were likely to be due to pre-existing factors i.e. obesity and diabetes. An identical study undertaken across 6 countries evaluated the tolerability of GLP-1 agonists in pregnant women and their effects on fetal outcomes (91). Study reported women with pregestational diabetes unveiled higher risks of cardiac malformations in the fetus, due to diabetes rather than the drug itself. Additionally, the prevalent use of GLP-1 agonists was highlighted emphasizing the need for more research in future (91).

An observational cohort study involving 168 pregnant women exposed to GLP-1 receptor agonists during the first trimester found a 2.6% prevalence of major birth defects, comparable to 2.3% in the diabetic reference group. While prior literature cites a 5–10% defect rate among women with pregestational diabetes, this study did not report any significant pregnancy losses following the use of GLP-1RA (92).

A systematic review executed on animal and human studies alongside case studies (93) demonstrated a dearth of human evidence on the excretion of GLP-1 agonists in breast milk, even though GLP-1 agonists, being large peptides, could be precisely present in human milk, although the exact amount is unclear (89). However, skeletal deformities and growth defects were prominently seen in rat models with no conspicuous findings in humans (93).

In addition to aforementioned properties of GLP-1 agonists, it can also reduce hypertension by activating and phosphorylating endothelial nitric oxide synthase pathways, thus increasing the bioavailability of nitric oxide which promotes vasodilation. Owing to these effects, liraglutide, a GLP-1 analog, has been studied as a potential drug for the treatment of pre-eclampsia, a pregnancy specific disorder, defined as new-onset hypertension after 20th week of gestation accompanied by proteinuria, as it is found to have anti-inflammatory and antioxidative effects (89). Pre-eclampsia has emerged as a notifiable cause of maternal and fetal morbidity and mortality (94).

Placental ischemia, as the causative factor for preeclampsia, leads to dysfunction of maternal vascular endothelium causing reduced synthesis of vasodilators and increased synthesis of endothelin, a potent vasoconstrictor. Production of pro angiogenic factors such as PIGF and VEGFZ and reactive oxidative species also plays a role in endothelial disruption (95). All these effects accentuate total peripheral resistance and impaired renal function raising blood pressure (95).

A study conducted on rat model demonstrated that liraglutide effectively reduced the mean arterial pressure, a cardinal feature for preeclampsia, leading to decreased blood urea nitrogen (BUN) and creatinine via renal vasodilation; however, it negatively affected the fetal weight, yet it was considered negligible due to liraglutide’s known effect of suppressing appetite (94). Moreover, liraglutide also displayed a boosted effect on levels of nitric oxide in mesenteric vessels and protection against endothelial dysfunction by increased production of plasminogen activator inhibitor type 1(PAI-1) and vascular adhesion molecule (VAM) (96).

As GLP-1 agonists have garnered attention for management of obesity due to their effect on reducing insulin sensitivity, they are now being considered a therapeutic option for the treatment of PCOS (polycystic ovarian syndrome), one of the most common causes of subfertility, diagnosed via Rotterdam’s criteria: oligomenorrhea, hyperandrogenism and polycystic ovaries (97). A statistical analysis assimilated various studies which insisted that GLP-1 agonists play a pivotal role in regulating menstrual cycle, ameliorating pregnancy rates and addressing infertility, a key concern in PCOS, though these benefits appeared to be temporary as drugs were only prescribed for 12 weeks and IVF pregnancies were unaffected. Thus, creating a void to conduct future studies to examine longer duration treatment to check their prolonged effects (98).

A comparative study conducted between GLP-1RA + Metformin in contrast to cyproterone acetate/ethinylestradiol + Metformin in overweight PCOS women, conducted over a period of 12 weeks, implied that GLP-1 RA+Metformin was superior in reducing the waist circumferences as well as preserving blood sugar levels by improved HbA1c levels and regulating menstrual cycle. Conversely, CPA/EE showed a stronger effect to reduce androgens levels (99). Similarly, a homogenous trial compared GLP-1RA against a placebo in PCOS women, demonstrating the significance of GLP-1RA analogs liraglutide and semaglutide, specifically. The results proved their efficacy by diminishing total testosterone and serum triglycerides, directly impacting the waist circumference and BMI (100), hence proving to be reliable therapy for PCOS.

There is a significant gap in research concerning the use of GLP-1 agonists during pregnancy, due to of lack of human studies as well as lack of data on their effects on lactation and fetal outcomes. More studies conducted with female rodent models and human subjects are needed to address these issues to ensure better maternal and fetal outcomes.

### 4.8 Effects on male reproductive system

GLP-1RAs may be very helpful in treating male infertility, especially in individuals who are obese and diabetic. Due to the expression of GLP-1 receptors in testicular cells, they can raise testosterone levels and increase sperm motility and metabolism (101). Also, they have a favorable impact on Sertoli cell metabolism, which is important in the production of sperm (102).

The effects of liraglutide on male obesity-associated functional hypogonadism (FH) are examined in this study. They designed a 16-week prospective randomized open-label study with 30 men, with BMI 41.2 ± 8.4 kg/m2 and aged 46.5 ± 10.9 years. An average of 7.9 ± 3.8 kg of weight was lost as a result of liraglutide medication, which also markedly improved general health and sexual performance. While luteinizing hormone (LH) and follicle-stimulating hormone (FSH) increased significantly (P < 0.001), total testosterone levels increased by+2.6 ± 3.5 nmol/L. On the other hand,0.9 ± 4.5 kg less weight was lost with testosterone replacement therapy (TRT) and +5.9 ± 7.2 nmol/L more advantageous for males with FH associated with obesity if lifestyle changes are unsuccessful (103). GLP-1 receptors are present in Sertoli and Leydig cells, which means that these drugs have the potential to directly affect testicular function in addition to improving hormonal balance. Studies show that GLP-1RAs enhance insulin production, motility, and sperm metabolism, all of which can result in improved sperm parameters. Furthermore, there is a correlation between improvements in sperm count and concentration and weight loss linked to GLP-1RA usage. However, more clinical research is required to validate these findings and investigate the long-term implications of using GLP-1RAs to treat male infertility because the precise pathways by which they affect male reproductive health are not entirely understood (101). Through a randomized, double-blind, placebo-controlled crossover trial, the effects of the GLP-1RA dulaglutide on sexual desire in healthy men were evaluated in a study. The study used the Massachusetts General Hospital-Sexual Functioning Questionnaire (MGH-SFQ) to assess sexual desire in 24 volunteers, ages 18 to 50. The results showed no discernible differences in hormone levels or sexual desire between dulaglutide and placebo, suggesting that dulaglutide had no negative effects on a healthy man’s ability to have sex. This gives certainty regarding its clinical application (104).

In order to close a knowledge gap regarding the relationship between metabolism and reproduction, the study investigates the effects of GLP-1 on the reproductive axis in healthy men (105) Enrolling eighteen fit male subjects ensured they were free of mental or physical illnesses and did not use prohibited substances. A single-blind, randomized, placebo-controlled crossover design was used in the study, which improves the validity of the results. The findings imply that GLP-1 may have an impact on reproductive hormones, pointing to possible treatment approaches for type 2 diabetes and obesity-related hypogonadism in males (105).

### 4.9 Effects on general anaesthesia

One of the major concerning side effects of GLP-1RA use in anesthesia are the complications of the GI tract presenting as delayed gastric emptying and elevated gastric residual content along with other symptoms (nausea, vomiting, abdominal distension and dyspepsia) (106). This leads to increase in gastric volumes and putting the patients at increased risk of aspiration during sedation (107).

The use of short acting GLP-1RA drugs has shown to improve glycemic control and promote insulin sectretion (53). However, these agents increase risk of intra-operative pulmonary aspiration according to a case report secondary to delayed emptying and presence of gastric residues despite fasting (108, 108)

To prevent these events, the recent recommendations from the American Society of Anesthesiology guidelines suggest discontinuing GLP-1 receptor agonists 1 day prior in patients on daily dosing and 1 week prior in patients on weekly injection dosing (108). Additionally, a precautionary step is to carry out an inexpensive and quick procedure like gastric ultrasound to look for any residues within the stomach prior to giving general anesthesia (109).

## 6 Adverse effects of GLP1-RA

In contrast to the advantages of GLP-1RA the analogues have gastrointestinal side effects distinctly nausea, emesis and reduced appetite with lower risk of hypoglycemia compared to other glucose lowering drugs (110). According to FDA, GLP-1RA had not shown evident risk of pancreatitis and pancreatic tumor (111). However, GLP-1RA are contraindicated for use in individuals with predisposition to MEN2 and medullary thyroid carcinoma (112). Additionally a meta-analysis found use of GLP-1RA was associated with higher risk of gallbladder and biliary disease with high doses for longer duration (113). These notable side effects can deter people from conforming to treatment plans.

## 7 Discussion

GLP-1 RAs have shown effects beyond their initial application in managing T2DM. GLP-1RA secondary to direct and mechanistic indirect effects on physiological systems have raised a need to further understand their potential in clinical practice. GLP-1RA, liraglutide and semaglutide are widely known for the therapeutic effects in management of T2DM for glycaemic control, Obesity for Weight Management along with Cardioprotective outcomes with influence on blood pressure, lipids and β -cell function (5). GLP-1RA have also shown neuroprotective effects with decreasing progression of neurodegenerative diseases and regulating neuronal activity (114). The newly discovered conjunctive use of GLP-1RA can improve clinical outcomes in patients with multiple chronic illnesses that have overlapping pathophysiology and help improve quality of life with early introduction.

Considering the expansive spread of GLP 1R throughout the systems in body and identifying the potential to modulate its effects with GLP-1RA is a paradigm shift in early intervention. GLP-1RA have shown to control HbA1C levels in patients with T2DM alongside alleviating the frequently existing comorbids, HTN and dyslipidaemia in combination with SGLT2 inhibitors (115). GLP-1RA as an independent class of drug is also noteworthy in reduction of weight in patients with and without poor glycaemic control with multiple trials backing the efficacy of agents including semaglutide and liraglutide (116). Cardiovascular outcomes have been investigated in clinical trials which suggested a reduction in cardiovascular events and mortality without risk of notable adverse effects. Meanwhile these agents have the potential to reduce major adverse cardiovascular events (MACE) in patient with T2DM as a drug class, individual drugs have different potency and effects they need tailored approach to align with patient treatment goals rather than generalized use (117). The GLP-1RA are limited to use in individuals without established cardiovascular disease which limits the use widely but provides its benefit for at risk population. Similarly, GLP-1RA have shown to have a neuroprotective effect including delay in Alzheimer’s and Parkinson’s disease onset as well progressions (118). Phase I and II clinical trials conducted have shown improved cognitive and motor function with different drugs from GLP-1RA group although the small sized and limited duration of trials have not provided a conclusive result to influence the practice, although with further advancement in ongoing trials effects observed may be noteworthy for intervention in future practice (119).

GLP-1RA have an amalgam of therapeutic effects assisting in providing combined intervention for diseases which are also complication of treatment of diabetes and anti-diabetic drugs. Use of GLP-1RA is protective and prevents bone fractures over a longer period of use in T2DM patients in a recent trial in 2020 liraglutide showed to have decreased bone resorption compared to placebo (120). Another use of GLP-1RA is also under study in ELAD (121), a phase II b clinical trial to provide strong evidence in using GLP-1RA as neuroprotective agents against Alzheimer’s Disease. These uses of GLP-1RA beyond the proven use in treatment and prevention of cardiovascular risk with obesity (110) as well as in diabetics add a new dimension to use them in early. The possibility of providing preventive regimen in multi system pathologies is a significant use of the GLP-1RA in improving disease outcomes and preventing morbidity.

## 8 Challenges in clinical application

Considering the enumerated benefits of GLP-1RA compared to other glucose-lowering drugs including the reduction in HbA1c and weight reduction benefits alongside decreased risk for hypoglycaemia (122) it is not cost effective if used in combination with another incretin (107). However, studies support GLP-1RA to be cost effective in comparison to insulin therapy as per the healthcare models in high income countries while the parameter for comparison between the two include the increased risk of hypoglycaemia and all-cause mortality alongside emergency room visits and hospitalization. This counteracts the high GLP-1RA purchasing, pharmacy and administration cost (123). The overall inflated cost adds to limitation of access to these drugs in resource poor nations (124).

## 9 Limitations of this review

This review does not cover the more expanded literature about the individual GLP-1RA drug administration, efficacy and side effects. A generalized GLP-1RA use has been explored across different clinical pathologies, although most of the literature narrated does not explore effects of GLP-1RA on diabetics and non-diabetics with concomitant renal and liver dysfunctions.

## 10 Future perspectives

In the recent era advancements seen in application of GLP-1RA is extensively appreciated to battle obesity and metabolic syndrome along with associated cardiovascular risks. However, a chasm is present for large scale studies to see the role GLP-1RA in neurological diseases including ocular pathologies, present studies are inconclusive about the implications in long term preventions in different populations. Alongside further clinical trials would benefit in learning about the role of GLP-1RA in gestational diabetes and implications on fetal and placental diseases. Responses to the less chartered topics will provide an insight into better and safer clinical practices.

In addition to finding the effects on multiple populations and pathophysiological impact of drug mechanisms, the future needs improved cost effective formulation and preparation to make GLP-1RA widely accessible. The potential to decrease burden of morbidity and mortality secondary to obesity, DM and cardiovascular diseases is a power GLP-1 RA can help harness to safe resources and improve quality of life as well as life span of populations at risk.

### 10.1 Future therapeutic effects

As advancements continue and overcome current gaps, GLP1-RAs are expected to become a drug of choice as a monotherapy or as combination therapy for a wide range of diseases. These include Alzheimer’s Disease where neuroprotective effects are under investigation followed by stroke under similar notion (125) Futhermore, in non-alcoholic fatty liver disease (NAFLD) they show evidence of improved liver histology and hepatic steatosis (126) in PCOS they may help in regulation of weight, reproductive and metabolic parameters simultaneously (127) GLP1-Ras are also under study with their ability to modulate the hemodynamic and inflammatory pathways to treat CKD (128) and Heart failure (129). Additionally the immunomodulatory and anti inflammatory potential might be prove useful in treating sepsis (130).

## 11 Conclusion

Glucagon-like peptide-1 receptor agonists are pivotal in managing T2DM and obesity, enhancing glycaemic control by boosting insulin secretion, reducing glucagon levels, delaying gastric emptying, and promoting satiety. They also reduce cardiovascular disease risk, as seen with semaglutide and liraglutide. Emerging roles include treating non-alcoholic fatty liver disease, preeclampsia, and neurodegenerative diseases like Alzheimer’s, due to anti-inflammatory and neuroprotective effects. GLP-1RAs may benefit glaucoma by reducing aqueous humour production. Despite their potential side effects like delayed gastric emptying, pancreatitis, and GI symptoms warrant tailored regimens. Future research will explore novel pathways and expand therapeutic applications.

## Glossary

GLP-1: Glucagon-like peptide-1
GLP-1R: GLP-1 receptor
GLP-1Ras: GLP-1R agonists
DPP4: Dipeptidyl peptidase 4
GRPP: Glicentin-related pancreatic polypeptide
cAMP: Cyclic adenosine monophosphate
T2DM: Type 2 Diabetes Mellitus
PKA: Protein Kinase A
RGNEF4: Rap Guanine Nucleotide Exchange Factor 4
IP3: Inositol triphosphate pathway
DAG: Diacylglycerol
CNS: Central Nervous System
BBB: Blood Brain Barrier
AD: Alzheimer’s Disease
PD: Parkinson’s Disease
ROS: Reactive Oxygen Species
BDNF: Brain-Derived Neurotrophic factor
LTP: Long Term Potentiation
Ex-4: Exendin-4
MPTP: 1-methyl-4-phenyl-1,2,3,6-tetrahydropyridine
CVOTs: CV outcome trials
CRP: C-reactive protein
SCALE: Satiety and Clinical Adipose Liraglutide Evidence
STEP: Semaglutide Treatment Effect in People with Obesity
BMI: Body Mass Index
VLDL: Very low-density lipoprotein
LDL: Low-density lipoprotein
ApoB48: Apolipoprotein B48
EAT: Epicardial adipose tissue
DPP-4i: Dipeptidyl peptidase-4 inhibitors
SGLT-2i: Sodium-glucose cotransporter-2 inhibitor
RGCs: Retinal Ganglion Cells
IOP: Intraocular pressure
DR: Diabetic Retinopathy
GDM: Gestational Diabetes Mellitus
PAI-1: Plasminogen activator inhibitor type 1
VAM: Vascular adhesion molecule
PCOS: Polycystic ovarian syndrome
FH: Functional hypogonadism
